# To the Editors of the Medical and Physical Journal

**Published:** 1804-10-01

**Authors:** 


### *To the Editors of the Medical and Physical Journal.*

GENTLEMEN,

DR. WALKER (No. 67, p. 242, Note) wishes to settle a proper or Classic term for Cow-pock, &c. *Vaccine*, French, is from the Latin: but it is objectionable, with all its derivatives; because, as Dr. W. observes, milk, butter, and cheese are all vaccine; and, indeed, the word more properly belongs to *them* than to the disorder. Milk is by Pliny adjectived with the word, *lac vaccinum*. Ainsworth, for want of higher authority at hand, must be referred to. *Vaccīne* alone, means *cowy*; to *vaccīnate*, is to cow, or to make of or like a cow; *vaccīnation*, is the making of or like a cow. *I* short, vaccīnate, cannot be.

Dr. Jenner's *variola vaccina* is good, but *two* words; and the term is wanted to be comprized in *one*. What Dr. W. calls the more happy term of Stokes, *vacciola*, cannot be admitted. It has a derivative form and termination; but whence is it derived? Not from *vaccia* or *vaccius*, for there are no such words; nor could there be from *vacca*, a cow: and if there could, *vacciola* would only mean, *a little cow*.

*Variolæ*, literally signifies, *small varieties*, that is, the spots or pits of small pocks with which the skin is varied. But the word is but a translation of *small-pox* by modern physicians. *Vara*, the disorder of *small-pocks* or *measles*, Ainsworth gives, from Littleton, as the authority of Pliny. This word also relates to the skin being *varied* by spots, pocks, or pits.

To find, therefore, one word to express the meaning of two, it may be a compound from two that are shortest. *Vaccæ-vara* put together, *vaccævara*, might express, *the cow-pox*. The word vaccævara would be what the Metricians or Grammarians would call a fourth Epitrite; that is, having the three first syllables long, and the fourth short. Thence might be, *vaccævarous matter*, (accent on the third syllable, on account of the quantity being long, and NOT on the second, like *cadāverous*). Thence also, *vaccævarátion*, and *vaccævarate*; accent on the fourth of the former, and on the third of the latter.

*Varus* and *varius*, Latin, being the same, but the first the original, the other derivative, *varation* must mean *variation*; so that *vaccævarátion* or *vaccævariátion* might either be used from *vaccævára* or *vaccævărĭă*.

*Obiter*,

*Obiter*, the vulgar and indelicate word *man-midwife*, should be hunted from the fronts of country laboratories. *Accoucher*, Fr. would soon be corrupted to *coucher*. Analagous to *obstetrix, fem.* is *obstitor*, in point of sense; and to *institor* it is analogous in form and composition. *Institor* is Horatian, and classical; why not, then, *Obstitor, ab obstando, vel obsistendo auxilii adferendi causa*?

*Brainular, scarlatina, caloric*, cum multis aliis, seem barbarisms.

I am, &c.

